# Tumor microenvironment changes after treatment with avelumab and immune-stimulating agent combinations in patients with advanced solid tumors

**DOI:** 10.21203/rs.3.rs-7775526/v1

**Published:** 2026-01-19

**Authors:** Nejla Ozirmak Lermi, Mohamed A Gouda, Jibran Ahmed, Xianli Jiang, Younghee Lee, Vakul Mohanty, Mohamed Derbala, Bettzy Stephen, Anuja Jhingran, Mohammad Moustafa Mohammad, Donghyun Joo, Alejandro Francisco-Cruz, Honglei Chen, Luis Acosta Calderon, Caddie Laberiano, Claudio Arrechedera, Renganayaki Pandurengan, Serdar Gurses, Yali Yang, Luisa M. Solis Soto, Jordi Rodon Ahnert, Ken Chen, Funda Meric-Bernstam, Eugene Jon Koay, Milind Javle, Cara Haymaker, Aung Naing

**Affiliations:** The University of Texas MD Anderson Cancer Center; The University of Texas MD Anderson Cancer Center; National Cancer Institute, National Institutes of Health; The University of Texas MD Anderson Cancer Center; The University of Texas MD Anderson Cancer Center; The University of Texas MD Anderson Cancer Center; The University of Texas MD Anderson Cancer Center; The University of Texas MD Anderson Cancer Center; The University of Texas MD Anderson Cancer Center; The University of Texas MD Anderson Cancer Center; The University of Texas MD Anderson Cancer Center; The University of Texas MD Anderson Cancer Center; The University of Texas MD Anderson Cancer Center; The University of Texas MD Anderson Cancer Center; The University of Texas MD Anderson Cancer Center; The University of Texas MD Anderson Cancer Center; The University of Texas MD Anderson Cancer Center; The University of Texas MD Anderson Cancer Center; The University of Texas MD Anderson Cancer Center; The University of Texas MD Anderson Cancer Center; The University of Texas MD Anderson Cancer Center; The University of Texas MD Anderson Cancer Center; The University of Texas MD Anderson Cancer Center; The University of Texas MD Anderson Cancer Center; The University of Texas MD Anderson Cancer Center; The University of Texas MD Anderson Cancer Center; The University of Texas MD Anderson Cancer Center

**Keywords:** Immunotherapy, Tumor microenvironment, OX40 agonists, CD137/4-1BB agonists, solid tumor

## Abstract

**Background:**

The use of immune checkpoint inhibitors (ICIs) has led to a paradigm change in cancer management. Many patients may have inherent primary resistance to ICIs or develop secondary resistance after initial response. The impact of using novel therapeutic combinations of checkpoint blockade (avelumab) with immune stimulating agonists such as anti-OX40 and/or anti-4-1BB on the tumor microenvironment and modulation of the immune response is an intriguing strategy to evaluate how these agents interact and whether the hypothetical rationale for combinations can be translated into augmentation of anti-tumor immunity in solid tumors.

**Methods:**

We performed whole exome sequencing (WES), bulk RNAseq, multiplex immunofluorescence (mIF) and chromogenic immunohistochemistry (IHC) on tumor tissue and flow cytometry of the peripheral blood to study longitudinal changes following the combination of avelumab with utomilumab (a 4-1BB agonist) (arm A), PF-04518600 (an OX40 agonist) (arm B), utomilumab and PF-04518600 (arm C) and utomilumab and radiotherapy (arm D) in phase I/II study (NCT03217747).

**Results:**

We observed low tumor mutation burden (TMB < 6) (median: 1.88), alteration of RTK-RAS, TP53, PI3K and WNT pathways across the cohorts. Mutations in *TP53*, *TTN* and *KRAS* (mostly p.G12C, p.G12D) genes and copy number variations (CNV) were found in *PIK3CA*, *CCNE1* and *KRAS*. Interferon gamma signaling pathway was enriched early on-treatment in tumors from patients with colorectal and pancreatic cancers in arm C. Patients deriving clinical benefit (CR/PR/SD ≥ 4 months) displayed higher T-cell frequencies at baseline (p = 0.0157), C1D15 (p = 0.0086), and C3D15 (p = 0.0070) than patients without clinical benefit.

**Conclusions:**

Our findings, though limited, highlight genomic differences between histologic subsets and outcome as well as the need for combination strategies that drive the recruitment and/or priming of anti-tumor T cells and address low immune permissive tumor states in patients with advanced solid tumors.

**Clinical trial registration:**

This clinical trial was registered on clinicaltrials.gov NCT03217747.

## Background

The use of immune checkpoint inhibitors (ICIs) has led to a paradigm change in cancer management; however, many patients may have inherent primary resistance to ICIs or develop secondary resistance after initial response. (1) Therefore, combination strategies have been proposed as an approach that could potentially improve not only response rates but also the durability of responses. (2) One combination strategy that has shown promising results in the preclinical setting is combining ICIs with OX40 or 4-1BB agonists which drive a costimulatory signal leading to improved immune response. (3–7) In a multi-arm phase I/II clinical trial (NCT03217747), we investigated the safety and efficacy of using avelumab in combination with utomilumab (previously known as PF-05082566, anti-41BB), Ivuxolimab (previously known as PF-04518600, anti-OX40), and/or radiation therapy in patients with advanced solid tumors (8, 9). Utomilumab and Ivuxolimab are examples of agonistic monoclonal antibodies that bind to 4-1BB/CD137 and OX40 respectively, resulting in immune activation and antitumor activity. (10, 11) Both drugs showed a tolerable safety profile when used in combination with preliminary tumor activity (8, 9, 12).

The impact of using different combinations of avelumab with OX40 and/or 4-1BB agonists on the tumor microenvironment and modulation of the immune response are interesting in the context of understanding how these agents interact and whether hypothetical rationale for combinations can be translated into augmentation of anti-tumor immunity. Herein, we report the results of correlative analysis of the clinical trial combining avelumab with utomilumab, ivuxolimab, and/or radiation therapy (NCT03217747).

## Methods

### Clinical Study Design

This was an open label single center phase I/II clinical trial (NCT03217747). Patients were eligible to participate if they had advanced or metastatic disease which was progressing on prior systemic therapy. Patients received treatment until disease progression or unacceptable toxicity. The trial included six arms with different combination regimens (A-E). In Arm A, patients received treatment with avelumab in combination with utomilumab (4-1BB/CD137 agonist). In Arm B, patients received treatment with avelumab in combination with Ivuxolimab (OX40 agonist). In arm C, patients received treatment with avelumab in combination with both utomilumab and Ivuxolimab. In arm D, patients received treatment with avelumab in combination with utomilumab and radiation therapy. In arm E, patients received treatment with avelumab in combination with Ivuxolimab and radiation therapy. In arm F, patients received treatment with avelumab in combination with utomilumab, Ivuxolimab, and radiation therapy. Based on reassessment of the study, the Joint Steering Committee amended the protocol deciding that Arms E and F were to be discontinued. Clinical data from this study was reported separately (9). Additional file 1 shows variations in timing of dosing for each agent according to each trial arm. In arm A, arm B, and arm C, maximum tolerated doses (MTDs) from other studies enrolling patients for treatment with the study drugs were used to directly enroll patients into dose expansion. Arm D had two phases: a dose de-escalation phase and an expansion phase. A standard 3 + 3 study design was used for dose de-escalation. The MTD was defined as the highest dose level with less than 2 patients with dose limiting toxicity (DLT) out of at least six patients in the cohort. Once the MTD was determined, additional patients were enrolled on the MTD expansion cohort for additional characterization of safety and response, and for correlative studies.

### Sample Collection

For patients enrolled in the MTD expansions of Arms A-C, tissue biopsies and blood samples were collected at baseline, between cycle 1 day 12 and 15 (prior to study drug dosing), prior to cycle 3 day 1 study drug dosing, and at the time of progression (if patient had an initial response of stable disease ≥ 4 months, partial response, or complete remission) to evaluate biological response or predictive markers in blood, tumor, and tumor environment and their relationships to drug exposure, clinical response, or other biologic response markers. For patients enrolled in the MTD expansion of Arm D, biopsies were collected at baseline, prior to cycle 3 day 1 dosing, and at the time of progression (if patient had an initial response of stable disease ≥ 4 months, partial response, or complete remission). In this cohort, blood samples were collected at baseline, prior to cycle 1 day 1 dosing, prior to cycle 1 day 15 dosing, prior to cycle 2 day 1 dosing, prior to cycle 3 day 1, and at the time of progression (if patient had an initial response of stable disease ≥ 4 months, partial response, or complete remission).

### WES data preparation and processing

We collected tissue samples at baseline, C1D15, C2D15 or PBMC samples of 46 patients with bile duct/cholangiocarcinoma (Arm A: n = 4 (13 samples); Arm B: n = 1 (4 samples); Arm C: n = 4 (14 samples)), cervical (Arm A: n = 6 (21 samples); Arm D: n = 3 (9 samples)), colorectal (Arm C: n = 3 (9 samples)), endometrial (Arm B: n = 5 (19 samples); Arm C: n = 2 (6 samples)), ovarian (Arm B: n = 6 (20 samples); Arm C: n = 1 (3 samples)), or pancreatic (Arm B: n = 7 (24 samples); Arm C: n = 4 (14 samples)) to perform WES. 37 (80%) of 46 patients showed no clinical benefit (SD < 4 months); 5 (11%) of 46 patients showed clinical benefits (CR/PR/SD ≥ 4 months); and 4 (9%) of 46 patients were not evaluable based on RECIST guidelines. gDNA was extracted by QIAamp^®^ DNA Mini Kit (Qiagen) then quantified using Quant-iT^™^ PicoGreen^™^ dsDNA Assay Kit (ThermoFisher SCIENTIFIC). Libraries were prepared with Agilent SureSelect Human All Exon v.4 probes (Agilent Technologies). The captured libraries were sequenced on Illumina NovaSeq 6000 platform for 2 × 150 paired end reads with an 8nt read for indexes using Cycle Sequencing v3 reagents (Illumina).

For WES data analysis, we aligned the WES capture deep-sequencing data to human reference assembly hg19 using BWA (13) and removed duplicated reads using Picard (14). We called single nucleotide variants (SNVs) and small indels using an in-house developed analysis pipeline (15). We called copy number alterations using a previously published algorithm (16). For WES, samples from patients in arm E and arm F as well as disease types with less than 5 patients and patients with missing baseline or PBMC samples were excluded from analysis. WES data analysis including somatic mutations, tumor mutation burden (TMB), copy number variation and pathway analysis (17) was visualized in R studio (18) with maftools (19), CNTools (20) and ggplot2 (21) packages.

### RNAseq data preparation and processing

We collected tissue samples at baseline, C1D15, C2D15 or end of treatment (EOT) from 44 patients with bile duct/cholangiocarcinoma (Arm A: n = 5 (11 samples); Arm C: n = 4 (10 samples)), cervical (Arm A: n = 6 (16 samples); Arm D: n = 3 (6 samples)), colorectal (Arm C: n = 3 (6 samples)), endometrial (Arm B: n = 5 (15 samples)), ovarian (Arm B: n = 6 (14 samples)), or pancreatic (Arm B: n = 7 (19 samples); Arm C: n = 5 (13 samples)) cancer to perform RNAseq. 33 (75%) of 44 patients showed no clinical benefit (PD/SD < 4 months); 6 (9%) of 44 patients showed clinical benefits (CR/PR/SD ≥ 4 months); and 5 (11%) of 44 patients were not evaluable based on RECIST guidelines. Total RNA was extracted from tissue samples using NORGEN Total RNA purification kit. RNA libraries were prepared using Agilent SureSelect Human All Exon v.4 probes (Agilent Technologies) and sequenced on Illumina NovaSeq 6000 platform for 2 × 150 paired end reads with an 8nt read for indexes using Cycle Sequencing v3 reagents (Illumina).

Raw RNAseq data was processed by an in-house RNAseq data analysis pipeline, which, among other tools uses the STAR aligner (22) to align raw reads to hg19 version of Human reference genome, featureCounts (23) to quantify aligned reads with to produce raw counts, and FastQC (24) and QualiMap (25) to evaluate quality of raw reads and feature counts. We used a BiocManager package DESeq2 (26) (with adjusted p-value < 0.05 with Benjamini Hochberg (BH) correction (27)) with vst normalization to identify differentially expressed genes between comparison groups. Batch effects were corrected from data using the limma package with its removeBatchEffect function (28). CibersortX (29) with absolute mode was performed to understand immune cell abundance between timepoints in diseases. In addition, Gene Set enrichment Analysis (GSEA) (30, 31) was performed. Results were visualized in R studio (18) using Enhanced Volcano (32), ComplexHeatmap (33) and ggplot2 (21) packages in R.

### Immunohistochemistry (IHC) data processing

We collected tissue samples at baseline, C1D15 and C2D15 from 71 patients diagnosed with bile duct/cholangiocarcinoma (Arm A: n = 1 (2 samples); Arm B: n = 1 (3 samples); Arm C: n = 4 (11 samples)), cervical (Arm A: n = 7 (23 samples)), colorectal (Arm C: n = 8 (16 samples)), endometrial (Arm B: n = 6 (16 samples); Arm C: n = 3 (8 samples)), ovarian (Arm B: n = 9 (22 samples); Arm C: n = 2 (5 samples)), pancreatic (Arm B: n = 8 (21 samples); Arm C: n = 10 (22 samples)), liver (Arm B: n = 1 (3 samples)), gastric (Arm C: n = 1 (1 samples)), uveal melanoma (Arm B: n = 1 (3 samples); Arm C: n = 1 (3 samples)), fallopian tube and adnexal (Arm B: n = 2 (5 samples)), renal (Arm C: n = 2 (7 samples)), and head and neck (Arm A: n = 4 (11 samples)) cancer to perform IHC and mIF assays. 49 (69%) of 71 patients showed no clinical benefit (PD/SD < 4 months); 12 (17%) of 71 patients showed clinical benefits (CR/PR/SD ≥ 4 months); and 10 (14%) of 71 patients were not evaluable based on RECIST guidelines.

Leica BOND^™^ RX autostainer and BOND^™^ Polymer Refine Detection kit (Leica Biosciences, DS9800) was used for the staining. Four-micron thick formalin-fixed paraffin embedded (FFPE) tissue slides were used for IHC. PD-L1, CD137(4-1BB), and OX40 were previously optimized and validated (34–36). The clone, catalogue number, vendor, RRID and dilution factor are summarized in the Additional file 2. All incubation steps were performed at room temperature with wash cycles in between each step unless otherwise stated. Bake and Dewax function were followed by a HIER step with Epitope Retrieval solutions 1 (ER1, pH6) for 20 minutes at 100°C. Next, tissues were incubated hydrogen peroxide block for 5 minutes, then with primary antibody for 15 minutes, followed by incubation with post-primary and polymer reagents for 8 minutes each. The antibody-antigen binding was visualized by brown DAB Refine stain for 10 minutes and counter-stained blue with Hematoxylin for 8 minutes. Tissue slides were then dehydrated offline, cleared in xylene, and cover-slipped using Cytoseal^™^ XYL solution.

PD-L1 was evaluated in malignant cells using a standard microscope approach and reported as percentage of malignant cells with any positive membrane expression. CD137 and OX40 were evaluated in malignant cells using a standard microscope approach and reported as percentage of malignant cells with any positive cytoplasmic or membrane expression. In addition, the number of CD137 and OX40 positive cells were quantified using digital image analysis (Aperio Image Toolbox^™^) in the entire tumor area and the results are expressed as cell densities from the analyzed area by mm2 (n/mm2).

### Multiplex immunofluorescence (mIF)

The multiplex immunofluorescence (mIF) assay is conducted on tissue slides using an automated Bond RX system. The process begins with slide preparation, including deparaffinization and antigen retrieval, to ensure optimal antigen exposure. Antibodies are then sequentially applied, each with a specific incubation period, followed by staining with TSA dyes for fluorescence-based detection.

The reagents, including OPAL dyes and detection buffers, are meticulously prepared to maintain staining integrity. The slides undergo multiple washing steps and counterstaining. After staining, the slides are mounted and imaged using quantitative software running on the slide scanner (Vectra Polaris), calibrated to ensure accurate fluorescence detection across whole-slide scans at low magnification.

The imaging process involves setting up scanning protocols, followed by the selection and analysis of regions of interest (ROIs) using Phenochart 1.0.12 software to assess marker expression. ROIs are then scanned at 20X magnification to produce multiplexed scanned images. Subsequent image processing is carried out using InForm 2.4.8 software, enabling detailed visualization and quantitative analysis of biomarker expression patterns. Comprehensive antibody information, including RRIDs, is provided in Additional file 3.

### Multiparameter flow cytometry data processing

We collected blood samples at baseline, C1D15 and C2D15 from 35 patients diagnosed with cervical (Arm A: n = 8 (24 samples)), colorectal (Arm C: n = 6 (14 samples)), endometrial (Arm B: n = 5 (13 samples); Arm C: n = 1 (2 samples)), ovarian (Arm B: n = 7 (19 samples); Arm C: n = 1 (3 samples)), pancreatic (Arm C: n = 3 (8 samples)), liver (Arm B: n = 1 (3 samples)), fallopian tube and adnexal (Arm B: n = 1 (3 samples)) and renal (Arm C: n = 2 (6 samples)) cancer to perform multiparameter flow cytometry. 19 (54.3%) of 35 patients showed no clinical benefit (PD/SD < 4 months); 12 (34.3%) of 35 patients showed clinical benefits (CR/PR/SD ≥ 4 months); and 4 (11.4%) of 35 patients were not evaluable based on RECIST guidelines.

Peripheral blood mononuclear cells (PBMCs) were washed with PBS containing 1% bovine serum albumin (BSA; catalog no. A8577, Sigma) and then blocked using 5% goat serum (catalog no. G9023, Sigma) in PBS/1% BSA for 30 min on ice to prevent nonspecific Fc receptor binding. For the myeloid panel staining, cells were incubated with a staining cocktail containing Brilliant Stain Buffer Plus (10 μl/test, catalog no. 566385, BD Biosciences), LIVE/DEAD Fixable Yellow Dead Cell Stain dye (1 μl/test, catalog no. L34968, Life Technologies) and a panel of antibodies targeting CD1c, CD3, CD11b, CD14, CD15, CD16, CD19, CD20, CD33, CD45, CD56, CD86, CD123, CD141, HLA-DR, PD-L1 (CD274) and PD-L2 (CD273) in PBS/1% BSA. Staining was performed for 30 min on ice in the dark. After incubation, cells were washed with PBS/1% BSA, fixed with 1% paraformaldehyde in PBS for 20 min at room temperature (RT) and washed again with PBS/1% BSA.

For T and NK panel staining, cells were incubated with a staining cocktail containing Brilliant Stain Buffer Plus, LIVE/DEAD Fixable Yellow Dead Cell Stain dye and surface antibodies targeting CD3, CD4, CD8, CD16, CD25, CD45, CD45RO, CD56, CCR7, CTLA-4 (CD152), OX40 (CD134), PD-1 (CD279), and 41BB (CD137). Following surface staining, cells were fixed and permeabilized using eBioscience Foxp3/Transcription Factor Staining Buffer Set (catalog no. 00-5523-00, Thermo Fisher Scientific) for 45 min on ice, then stained intracellularly with FOXP3 and Ki67 antibodies for 45 min at RT.

Samples were acquired using the BD Fortessa X20 flow cytometer and analyzed using FlowJo Software v.10.10.0 (Tree Star, Inc.). The associated antibodies, RRIDs and gating strategies are shown in Additional file 4 and Additional file 5: Fig S1.

### Statistical Test Analysis

Wald test, Wilcoxon signed rank test (two-sided) and Mann Whitney U test were performed to display statistical significance between groups.

## Results

**Figure 1:**
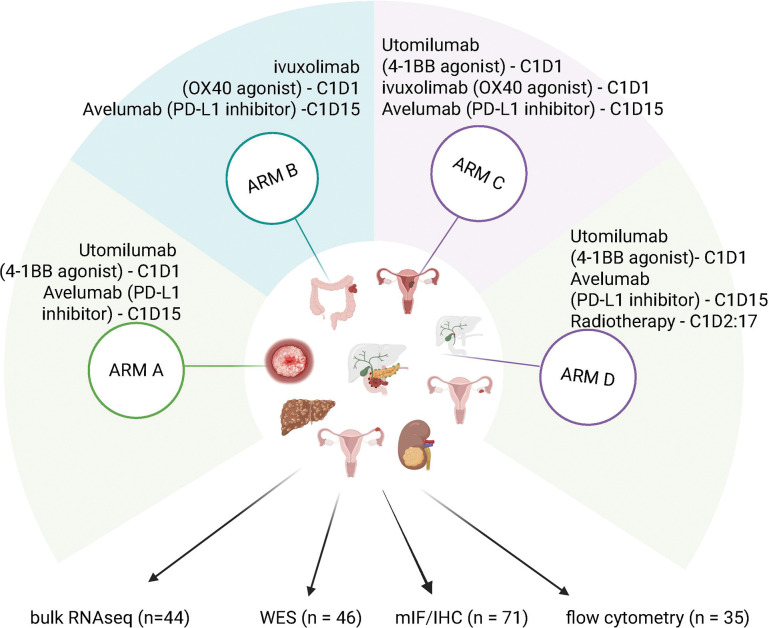
Experimental design of study. Multi-arm study with combination of avelumab with CD137/4-1BB and OX40 agonists. Patients were diagnosed with multiple solid advance level disease. Created by BioRender.com.

This phase I/II clinical trial (NCT03217747) enrolled patients with advanced or metastatic disease which was progressing on prior systemic therapy. Samples were available from a total of 98 patients with breast, bile duct/cholangiocarcinoma, cervical, colorectal, endometrial, ovarian, pancreatic, liver, uveal melanoma, melanoma, fallopian tube and adnexal, gastric, renal, gallbladder, ampullary and head and neck cancer diagnosis who were enrolled to the study of avelumab with CD137/4-1BB agonists (arm A) (n = 24), avelumab with OX40 agonists (arm B) (n = 28), avelumab with Cd137/4-1BB agonists and OX40 agonists (arm C) (n = 32) and avelumab with CD137/4-1BB agonists and radiation (arm D) (n = 14). Figure 1 illustrates the combinations, timing, and number of total samples available for correlative analysis by assay.

### Somatic variants and copy number variation analysis unveil resistant genes and signatures in avelumab combination therapy.

To understand somatic variant (SNV) reposition and copy number variations (CNV), we utilized WES data from different timepoints of 46 patients with bile duct/cholangiocarcinoma, cervical, colorectal, endometrial, ovarian, or pancreatic cancer that were treated based on arm A, B, C and D combinations (Fig. 1).

**Figure 2:**
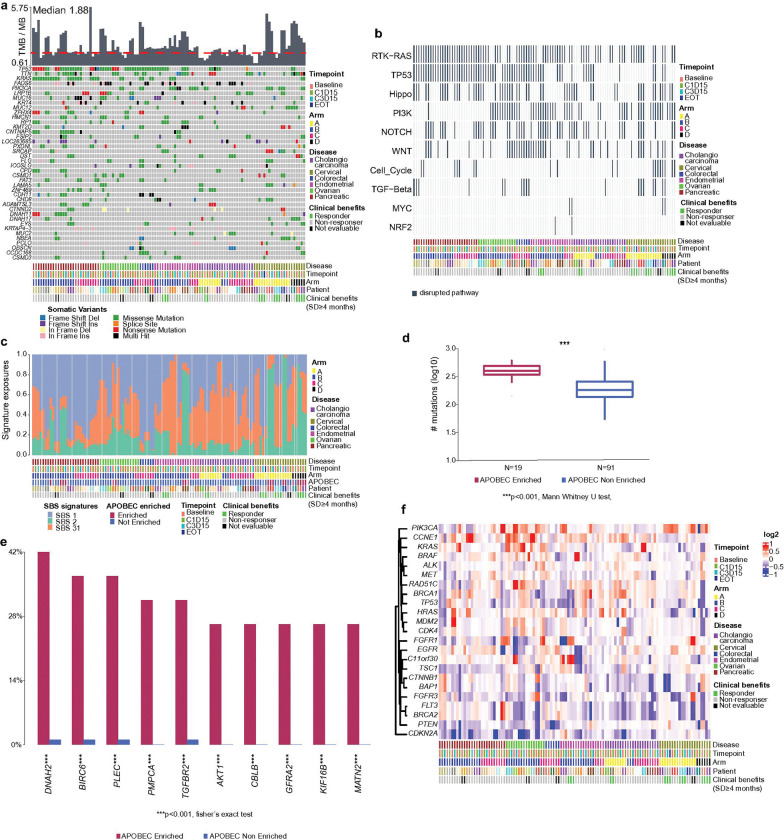
Somatic alterations at DNA in the combination therapy. **a,** Bar plot at top displays TMB of patients. Red dashed line represents median TMB of this cohort. Heatmap shows top highly mutated genes in patients. Each color represents a type of somatic variant in the gene. Non-mutated genes are labeled with gray color. Bar plot at bottom displays the clinical information of samples. **b,** Disrupted oncogenic pathways in samples. **c,** Bar plot displays COSMIC single base substitute (SBS) signatures in samples. Each color represents a SBS signature. **d,** Box plot represents differences of number of mutations between APOBEC enriched and non-enriched samples. The center of the box plot is denoted by the median, a horizontal line dividing the box into two equal halves. The bounds of the box are defined by the lower quartile (25th percentile) and the upper quartile (75th percentile). The whiskers extend from the box and represent the data points that fall within 1.5 times the interquartile range (IQR) from the lower and upper quartiles. **e,** Heatmap displays copy number variation (CNV) in selected genes. **f,** Bar plot shows differences of percentage of disrupted genes between APOBEC enriched and non-enriched samples.

Tumor mutation burden (TMB) was less than 6 SNV per million bases with a median of 1.88 in all included disease types (Fig. 2a). We observed an increase in TMB between C1D15 and C3D15 timepoints in pancreatic cancer patients (p = 0.031) (Additional file 5: Fig S2). *TP53*, *TTN*, and *KRAS* were frequently mutated in pancreatic, ovarian, and colorectal tumors; *PIK3CA* was mutated in endometrial; *IDH1*, *DNAH11* and *ZFHX4* were mutated in bile duct/cholangiocarcinoma; and *TGFBR2* was mutated in cervical cancer with missense mutations. *KRAS* mutations identified included p.G12A, p.G12C, p.G12D, p.G12R, p.G12V, p.G13D, p.Q61H and p.A146T. We observed the same mutations were detected in all timepoints in the same patient (Fig. 2a). We evaluated alterations in mutated oncogenic pathways using WES and observed disruptions in mostly RTK-RAS signaling, TP53, Hippo, PI3K, NOTCH and WNT signaling pathways (Fig. 2b).

We also analyzed COSMIC single base substitutions (SBS) signatures and APOBEC analyses. While we observed high SBS1 (clock-like mutational signatures due to correlation with age) signatures in pancreatic and colorectal cancer, SBS2 (activation of AID/APOBEC cytidine deaminases in cancer) signature was high in cervical cancer patients (Fig. 2c). We also detected SBS31 (related to chemotherapy treatment with platinum drugs) in most of the patients due to previous treatment strategies.

Given the observed high enrichment of SBS2 signature in cervical cancer, we performed APOBEC enrichment analysis and found that most of the cervical cancer patients had APOBEC enrichment (n = 5/9 patients) with significant high expression of *DNAH2*, *BIRC6* and *PLEC* in APOBEC enriched patients (Fig. 2c, d, e). CNV analysis reveals amplification of *PIK3CA*, *CCNE1*, *KRAS* and *RAD51C*; and deletion of *FGFR1*, *EGFR*, *CDKN2A* in ovarian cancer (Fig. 2f).

### Transcriptomic analysis displays changes in both tumor intrinsic and tumor extrinsic pathways and suggest an increase in immune evasion on-treatment.

**Figure 3:**
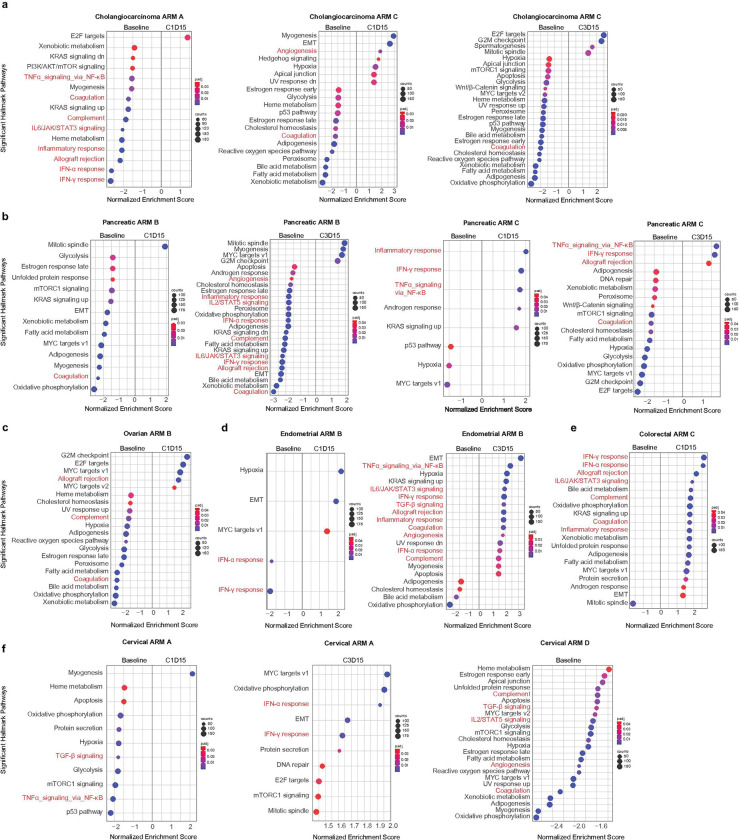
Hallmark pathway enrichment analysis. Bubble plots show significant differentially enriched pathways between timepoints **a,** in cholangiocarcinoma arm A and C**, b,** in pancreatic arm A and C, **c,** in endometrial arm B, **d,** in ovarian arm B, **e,** in colorectal arm C, **f,** in cervical cancer arm A and D.

To investigate pathway enrichment signatures of treatment resistance in the cohorts, we analyzed bulk RNA-seq data from different timepoints of 51 patients with bile duct/cholangiocarcinoma, cervical, colorectal, endometrial, ovarian, or pancreatic cancer that were treated based on arm A, B, C and D combinations. We observed significant (adjusted p-value < 0.05, Benjamini-Hochberg (BH) correction) high enrichment of tumor-intrinsic pathways that were related to tumor development and immune evasion at C1D15 and/or C3D15 such as E2F targets, G2M checkpoint and MYC targets V1 signaling pathways in bile duct/cholangiocarcinoma (arm A and C) (Fig. 3a), pancreatic (arm B) (Fig. 3b) and ovarian (arm B) (Fig. 3c). Patients with endometrial cancer (arm B) displayed high enrichment at C1D15 in hypoxia, epithelial-mesenchymal transition, and MYC targets V1 signaling pathways. By C3D15, an enrichment of tumor-extrinsic pathways such as IFN-α and IFN-γ response, TNF-α signaling via NFKβ and IL-6-JAK-STAT signaling pathways were observed (Fig. 3d). Tumor-extrinsic pathways such as IFN-α, IFN-γ, TNF-α signaling via NFKβ and IL-6-JAK-STAT signaling pathways were enriched at C1D15 and/or C3D15 in colorectal cancer (arm C) (Fig. 3e) and cervical cancer (arm A and D) (Fig. 3f).

**Figure 4:**
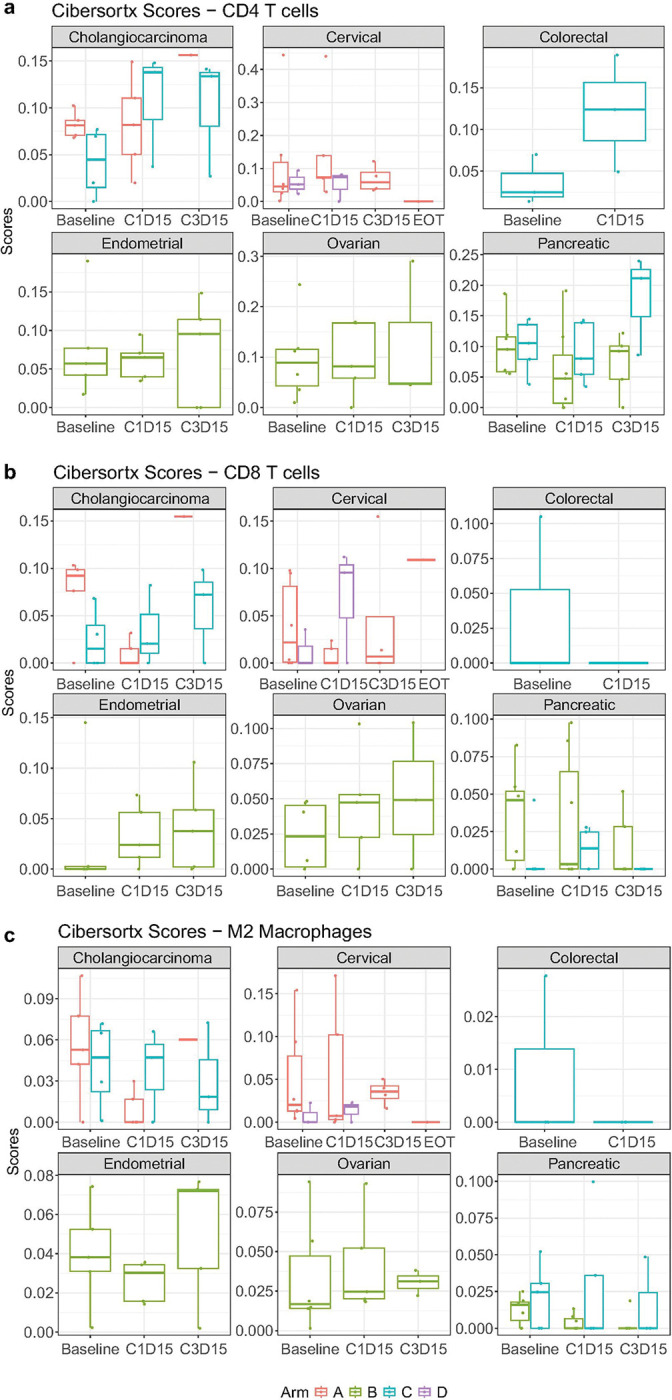
Inferred immune cell type abundance. Box plots display differences between timepoints and arms **a,** in CD4 T cells, **b,** in CD8 T cells, **c,** in M2 macrophages. The center of the box plot is denoted by the median, a horizontal line dividing the box into two equal halves. The bounds of the box are defined by the lower quartile (25th percentile) and the upper quartile (75th percentile). The whiskers extend from the box and represent the data points that fall within 1.5 times the interquartile range (IQR) from the lower and upper quartiles. Any data point outside this range is considered an outlier and plotted individually.

In addition, we assessed inferred immune cell type abundance between arms and timepoints by performing CibersortX with absolute mode and investigated the change in abundance of CD4 T cells (activated by OX40 agonists), CD8 T cells (activated by CD137/4-1BB agonists) and M2 Macrophages (inhibited by avelumab) to understand the impact of the ICI combinations on these immune subsets in the TME. We observed similar inferred CD4 T cells between baseline and C1D15 in arm A, and more CD4 T cells at C1D15/C3D15 than baseline in pancreatic cancer but no change in other disease types in arm B, and less or no change in CD4 T cells at C1D15/C3D15 than baseline in the combination of OX40 and 4-1BB agonists with avelumab (arm C) (Fig. 4a). We detected a reduction of inferred CD8 T cells in cholangiocarcinoma (arm A) at C1D15 and an increase of inferred CD8 T cells in arm B and cervical cancer (arm D) at C1D15/C3D15 compared to baseline (Fig. 4b). Finally, we identified a trending reduction of M2 macrophage cell abundance at C1D15/C3D15 in bile duct/cholangiocarcinoma (arm A) and pancreatic (arm B) cancer, but we did not observe this trend in other disease types (Fig. 4c). The changes in CD4 and CD8 T cells and M2 macrophages were limited due to sample size, and our conclusions are based on descriptive statistics rather than formal statistical inference.

### Clinical benefit is associated with a higher presence of CD8 + T cells in the TME prior to therapy.

To evaluate the relationship between immune cell types and clinical outcome, we analyzed the abundance of multiple immune cell populations using chromogenic immunohistochemistry (IHC) and multiplex immunofluorescence (mIF) from 71 patients diagnosed with bile duct/cholangiocarcinoma, cervical, colorectal, endometrial, ovarian, pancreatic, liver, gastric, uveal melanoma, fallopian tube and adnexal, renal, and head and neck cancer across different timepoints (Baseline, C1D15, and C3D15). Patients were stratified into clinical benefit (CR/PR/SD ≥ 4 month) and no clinical benefit (PD/SD ≤ 4 month), and statistical comparisons (Wilcoxon rank sum test (two-sided)) were performed between groups to evaluate differences by time point.

**Figure 5:**
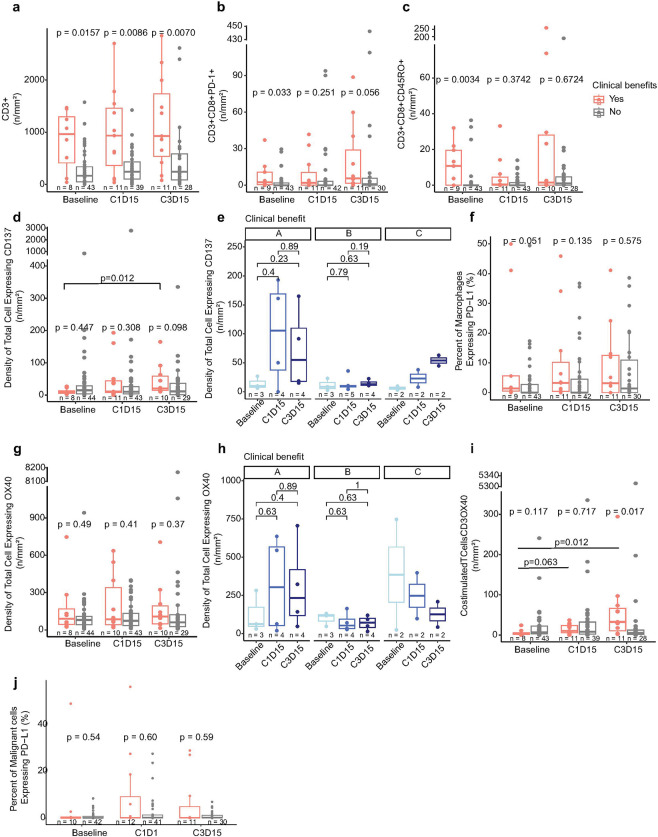
mIF and IHC demonstrates higher infiltration of T cells and expression of CD137/4-1BB and OX40 relates with clinical response. **a,** Number of T cells in patients with versus without clinical benefits at baseline, C1D15, C3D15. **b,** Number of Cytotoxic T cells expressing PD-1 in patients with versus without clinical benefits at baseline, C1D15, C3D15. **c,**Number of effector/memory cytotoxic T cells in patients with versus without clinical benefits at baseline, C1D15, C3D15. **d,** Density (%) of total cell expressing CD137/4-1BB in patients with versus without clinical benefits at baseline, C1D15, C3D15. **e,** Density (%) of total cell expressing CD137/4-1BB in patients with clinical benefits at baseline, C1D15, C3D15 stratified by cohort. **f,** Percent of macrophages expressing PD-L1 in patients with versus without clinical benefits at baseline, C1D15, C3D15. **g,**Density (%) of total cell expressing OX40 in patients with versus without clinical benefits at baseline, C1D15, C3D15. **h,** Density (%) of total cell expressing CD137/4-1BB in patients with clinical benefits at baseline, C1D15, C3D15 stratified by cohort. **i,** Number of T cells expressing OX40 in patients with versus without clinical benefits at baseline, C1D15, C3D15. **j,**Percent of malignant cells expressing PD-L1 in patients with versus without clinical benefits at baseline, C1D15, C3D15. Wilcoxon rank sum test (two-sided) was used. Nominal p values were shown in the plots. The center of the box plot is denoted by the median, a horizontal line dividing the box into two equal halves. The bounds of the box are defined by the lower quartile (25th percentile) and the upper quartile (75th percentile). The whiskers extend from the box and represent the data points that fall within 1.5 times the interquartile range (IQR) from the lower and upper quartiles. Any data point outside this range is considered an outlier and plotted individually.

The patients with clinical benefit exhibited a significantly higher infiltration of total T cells (CD3+) compared to those without clinical benefit at baseline (p = 0.0157), C1D15 (p = 0.0086), and C3D15 (p = 0.0070) (Fig. 5a). Cytotoxic T cells expressing PD-1 (CD3 + CD8 + PD-1+; Fig. 5b) and Effector/Memory Cytotoxic T cells (CD3 + CD8 + CD45RO+; Fig. 5c) were higher in the clinical benefit group at baseline (p = 0.033, p = 0.0034), but this difference did not reach statistical significance on-treatment. Overall, no significant difference was observed between timepoints, suggesting initial presence but not expansion of total cytotoxic T cells was associated with response.

Higher densities of cells expressing CD137/4-1BB at baselines and on treatment tumor samples were associated to clinical benefit in cohorts A, C and D (Fig. 5d). We observed an increase in densities of cells positive for CD137/4-1BB in clinical benefit group were observed in cohorts A and C, although this change was not statistically significant. (Fig. 5e).

OX40, a marker associated with T cell activation, was similarly expressed in clinical benefit group across timepoints (Fig. 5g). We did not observe an increase of densities of OX40 positive cells in clinical benefit group of cohorts B and C (Fig. 5h). However, co-stimulated T cells (CD3) and OX40 were elevated in clinical benefit group across timepoints (p = 0.063, p = 0.012), and significantly higher compared to clinical non-benefit at C3D15 (p = 0.012) (Fig. 5i). The clinical benefit group also exhibited higher baseline abundance of Macrophages expressing PD-L1 (p = 0.051), suggesting they were more sensitive to PD-L1 blockage (Fig. 5f). Patients did not display differences in abundance of malignant cells expressing PD-L1 (p = 054, p = 0.60, p = 0.59) (Fig. 5j).

### Systemic immune activation was not observed across analyzed cohorts.

We investigated changes in the frequency and activation of T, NK cell and myeloid cell populations in the peripheral blood using flow cytometry in 35 patients diagnosed with cervical, colorectal, endometrial, ovarian, pancreatic, liver, fallopian tube and adnexal or renal cancer across different timepoints (Baseline, C1D15, and C3D15). We did not observe any differences between diseases, timepoints or clinical benefit in percentage of CD4 and CD8 T cells (Additional file 5: Fig S3 a, b, c, d). In addition, we investigated the percentage of CD137/4-1BB, OX40 and PD1 expression by CD4 and CD8 T cells. We did not see any elevation in CD137/4-1BB, OX40 or PD1 at C1D15 and C3D15 in any arms (Additional file 5: Fig S3 e, f, g, h, I, j).

## Discussion

We reported the correlative data analyses of Phase I/II clinical trial (NCT03217747) with combination of avelumab (PD-L1 inhibitor) with utomilumab (4-1BB/CD137 agonist), ivuxolimab (OX40 agonist), and/or radiation therapy (NCT03217747) in advanced or metastatic diseases. Patients in the trial showed resistance to this multi-arm combination therapy. We displayed interrupted genes and oncogenic pathways at DNA level, enrichment of Hallmark pathways and inferred immune cell type abundance at RNA level, specific protein expressions with multiplex immunofluorescence (mIF) and immunohistochemistry (IHC) in tissue and with flow cytometry in blood circulation at Baseline, C1D15 and C3D15.

We observed low TMB (< 6 mutations per megabases) in all patients, and *KRAS* mutations at p.G12A, p.G12C, p.G12D, p.G12R, p.G12V, p.G13D, p.Q61H and p.A146T, alteration of RTK-RAS, TP53, Hippo and PI3K oncogenic pathways can be results of resistance to therapy in these patients (37). While patients had different gene mutations between disease types, they resisted the therapy. High exposure of these two SBS signatures were consequences of errors in DNA replication, and they involved in tumor growth and patient prognosis (38). Previously researchers displayed the association of APOBEC expressions and poor prognosis in cervical cancer (39).

Treating patients with OX40 and CD137/4-1BB agonists in the combinations aimed to increase T cell activation prior to avelumab treatment to improve the response rate to PD-L1 blockade treatment. We did not observe enrichment of IFN-γ response pathway at C1D15 timepoint in patients with cholangiocarcinoma in arm A and C, pancreatic in arm B, endometrial in arm B, ovarian in arm B and cervical cancer in arm A and D. However, patients with colorectal and pancreatic cancer in arm C displayed high enrichment of IFN-γ response pathway. Interestingly, patients with endometrial cancer in arm B and cervical cancer in arm A enriched higher IFN-γ response pathway at C3D15 after avelumab treatment. These findings suggested that OX40 and CD137/4-1BB agonists did not work similarly to activate T cells in all patients.

Furthermore, we exhibited the abundance of CD4 T cells based on inferred immune cell type abundance analysis to understand whether OX40 against increased the CD4 T cell population and found that abundance of CD4 T cells increased in cholangiocarcinoma and colorectal cancer in arm C, but not other patients in arm B or C as a trend. We observed a reduction in CD8 T cells in all patients at C1D15 in arm A, even though these patients received 4-1BB agonists at C1D1. Inconsistent elevation of CD4 and CD8 T cells at C1D15 supported the unpredictable IFN-γ response pathway enrichment. Although 4-1BB agonist treatment did not increase CD8 T cell population in patients of arm A, we displayed reduction of M2 macrophages at C1D15 cholangiocarcinoma patients.

Later, we investigated the differences between clinical benefitted and non-benefitted patients and showed that patients with clinical benefit exhibited a higher abundance in total lymphocytes, cytotoxic T cells, OX40 expression and PD-L1 expressing-Macrophages prior to treatment. The elevated CD137/4-1BB and OX40 expression suggested that immune activation and expansion were already present prior to therapy in the clinical benefit group.

This study has limitations. The number of patients in each arm and disease type at different timepoints were not enough to investigate differential immune or tumor changes by IO combination and effects of radiation. In addition, data was not available from all-time points in all patients; thus, pairwise data analysis was not performed, and data analyses should be considered highly exploratory.

## Conclusions

Our findings confirm that OX40 and CD137/4-1BB were not highly expressed in advanced solid tumor patients as we observed in previous studies to eliminate resistance to avelumab treatment. Future efforts to improve combination therapies using immune agonists for advanced solid tumors should prioritize a deeper understanding of the mechanistic pathways and cellular interactions that define the dynamic tumor microenvironment. Critical areas include clarifying the functions of immune cells, stromal elements, extracellular matrix, and soluble mediators that together drive tumor progression, immune evasion, and therapeutic resistance. Harnessing advanced technologies such as single-cell multi-omics, spatial transcriptomics, and microfluidic models will be pivotal for mapping TME heterogeneity and uncovering actionable targets. These mechanistic insights can guide the rational design of combination strategies that transform immunosuppressive or “cold” TMEs into immunepermissive states, amplify antitumor activity, and overcome resistance to existing therapies. An integrated, interdisciplinary approach to decoding TME complexity will accelerate the development of more effective, adaptable, and personalized cancer treatments.

## Supplementary Material

Supplementary Files

This is a list of supplementary files associated with this preprint. Click to download.

• Additionalfile1DrugDosing.xlsx

• Additionalfile2IHCantibodies.xlsx

• Additionalfile3mIFantibodies.xlsx

• Additioonalfile4FlowcytometryAntibodylist.xlsx

• AdditionalFile5.pdf

## Data Availability

Data can be available upon reasonable request from corresponding author.

## Code Availability Statement:

Data analyses are performed using open-source public tools and methods section includes packages and versions of tools that are used in the data analysis. Scripts can be available upon reasonable request from corresponding author.

## Abbreviations

APOBEC: Apolipoprotein B mRNA Editing Catalytic Polypeptide-like
BIRC6: Baculoviral IAP Repeat Containing 6
BH: Benjamini Hochberg
C1D15: Cycle 1 day 15
C2D15: Cycle 2 day 15
CCNE1: cyclin E1
CCR7: C-C Motif Chemokine Receptor 7
CD: Cluster of Differentiation
CD137/4-1BB: Tumor necrosis factor ligand superfamily member 9 (TNFSF9)
CNV: Copy number variation
COSMIC: Catalogue Of Somatic Mutations In Cancer
CR: Complete remission
CTLA-4: Cytotoxic T-lymphocyte associated protein 4
DLT: Dose limiting toxicity
DNAH: dynein axonemal heavy chain
dsDNA: Double stranded deoxyribonucleic acid
EGFR: epidermal growth factor receptor
FFPE: Formalin-fixed paraffin embedded
gDNA: Genomic deoxyribonucleic acid
GSEA: Gene Set enrichment Analysis
ICIs: Immune checkpoint inhibitors
IDH1: Isocitrate dehydrogenase
IHC: Immunohistochemistry
IFN: Interferons
KRAS: Kristen Rat Sarcoma Viral oncogene homolog
mIF: Multiplex immunofluorescence
MTDs: Maximum tolerated doses
NFKβ: Nuclear factor kappa-light-chain-enhancer of activated B cells
OX40: Tumor necrosis factor receptor superfamily, member 4 (TNFRSF4)
padj: Adjusted p-value
PBMC: peripheral blood mononuclear cells
PD-L: Programmed death-ligand
PI3K: Phosphoinositide 3-kinases
PIK3CA: phosphatidylinositol-4,5-bisphosphate 3-kinase catalytic subunit alpha
PR: Partial response
RECIST: Response Evaluation Criteria in Solid Tumors
ROI: regions of interest
RNAseq: RNA sequencing
RRID: Research Resource Identifiers
RTK-RAS: Receptor Tyrosine Kinase-RAS
SBS: Single base substitutions
SD: Stable disease
SNV: Single nucleotide variants
TGFBR2: Transforming growth factor beta receptor 2
TMB: Tumor mutation burden
TME: Tumor microenvironment
TNF: Tumor necrosis factor
TTN: Titin
TP53: Tumor protein p53
VST: Variance stabilizing transformation
WES: Whole exome sequencing
WNT: Wingless-type MMTV integration site family
ZFHX4: Zinc finger homeobox 4
